# Experimental evaluation of the use of starch and carboxymethylcellulose in the prevention of intraperitoneal adhesions in hernia surgery with coated meshes

**DOI:** 10.1590/acb383323

**Published:** 2023-09-18

**Authors:** Paulo Vicente dos Santos, Elcio Shiyoiti Hirano

**Affiliations:** 1 Universidade Estadual de Campinas – Postgraduate Program in Surgical Science – Campinas SP – Brazil.

**Keywords:** Tissue Adhesions, Surgical Mesh, Hernia, Implants, Experimental

## Abstract

**Purpose:**

Laparoscopic hernia repair involves a risk of adhesion between coated mesh and viscera. Plant polysaccharides such as starch and carboxymethylcellulose (SC) make up a product that acts as a barrier and prevents intraperitoneal adhesions. This study aimed to evaluate whether topical SC can also reduce adhesions between mesh and intra-abdominal organs.

**Methods:**

Forty rats each received placement of two intraperitoneal mesh fragments, one on each side of the abdominal wall. Randomly, 20 animals received SC on the right and other 20 on the left, leaving the contralateral side as a control. Fourteen days after the surgery, the animals underwent an additional laparotomy in which macroscopic analysis was performed.

**Results:**

As for the percentage of the mesh area affected by adhesion, one (2.6%) animal had > 75% adhesion on the experimental side, and 11 animals (28.9%) on the control side. The adhesion intensity score showed firm adhesions in three (7.9%) animals on the experimental side and nine (23.7%) on the control side.

**Conclusions:**

The use of SC decreased the intensity of adhesions and the surface area of the mesh affected by adhesions. There was no negative interference of the product in the incorporation of the mesh into the abdominal wall.

## Introduction

The exact global incidence of incisional hernias is uncertain, with 2–20% of all laparotomies developing incisional hernias, especially midline laparotomies^1^,^2^. The recurrence of these hernias in cases of complex repair ranges between 20–30%, with a 30% recurrence after a second repair in cases of a new recurrence^3^. These numbers are increasing due to the increasing number of patients undergoing laparotomies and major surgeries, older patients with connective tissue problems, and patients operated on with risk factors for hernia formation, such as obesity and diabetes^4^,^5^.

Laparoscopic surgeries are associated with a lower rate of early complications, such as surgical site infection, seroma, and bleeding^6^, with a shorter duration of hospital stay and fewer hernia recurrences^7^. Also, other inherent advantages are reduced postoperative pain, earlier recovery, and favorable aesthetic effects^8^,^9^.

Despite these advantages, laparoscopic repairs using the intraperitoneal onlay mesh (IPOM) technique expose the patient to the risks of mesh in direct contact with intra-abdominal organs and structures. Even when using meshes which supposedly provide a protective barrier, there are reports of complications secondary to ileus, adhesions, mesh infections, abscesses, fibrosis, chronic pain, and even intestinal obstruction, and enteric fistulas^9^-^13^. In addition, possible long-term complications of this technique have not been fully elucidated^14^.

Adhesions that increase these potential risks occur due to foreign body reaction secondary to the inflammatory reaction generated by the presence of a prosthesis inside the abdominal cavity and in contact with intracavitary organs^10^,^11^,^15^,^16^. Some products available on the market can reduce adhesion formation whether between organs or intraperitoneal structures^17^-^21^ or between the mesh and intra-abdominal structures^22^,^23^. In this context, plant polysaccharides such as starch and carboxymethylcellulose (SC) make up a product that acts as a hemostatic and mechanical barrier and prevents intraperitoneal adhesions^24^,^25^.

The objective of this study was to evaluate whether topical SC can reduce adhesions between the mesh and intra-abdominal organs after IPOM hernia repair in an experimental model, in addition to the already known effect of preventing intraperitoneal adhesions.

## Methods

### Ethical approval

This research project complied with the rules of the National Council for Animal Experimentation and was approved by the Animal Ethics Committee of Universidade Tiradentes (no. 011120).

### Intraperitoneal mesh

The Symbotex mesh is a synthetic, nonabsorbable mesh made of three-dimensional polyester monofilaments coated with bioabsorbable collagen film on its visceral side. Polyester is the result of the reaction of alcohol with carboxylic acid and is a strong, durable, and hydrophilic material. It has pores of 2.3 – 3.3 mm and 66 g/m^2^ and has multiple filaments^10^. The mesh was donated by the company.

### Anti-adhesion product

Adhesion STP (ASTP) is a natural product made by the plant polysaccharides SC, which have hemostatic and anti-adhesion properties. Carboxymethylcellulose is a low-molecular-weight and water-soluble cellulose product. It reduces adhesion and fibrosis formation by creating a film that works as a mechanical barrier, acting as a healing reorganization agent^26^. Starch is derived from plants and acts by dehydrating the blood. It also has a hemostatic action when associated with carboxymethylcellulose^27^. In addition, this combination stimulates the production of tissue plasminogen activator by macrophages, which results in the conversion of plasminogen into plasmin, with consequent fibrinolytic action and decreased fibrin and adhesion formation^25^. Carboxymethylcellulose is quickly metabolized by human enzymes, and starch acts for approximately 40 days, generating a low risk of granulomatous reaction due to the short absorption period^26^,^28^.

### Experimental design

A total of 40 Wistar male rats weighing between 250 and 350 g were used. The animals were housed in the bioterium, with a natural light-dark cycle, adjusted environmental temperature and humidity, and fed and watered ad libitum, both before and after surgery.

The animals underwent laparotomy with placement of two intraperitoneal mesh fragments, one on each side of the abdominal wall. Randomly, 20 animals received SC in the abdominal cavity on the right, and other 20 animals received SC on the left, leaving the contralateral side as a control (with mesh and without SC). The experiment used two 3-g bottles of the product, totaling 6 g, equally divided among the 40 animals analyzed using a precision scale.

Fourteen days after the surgery, the animals underwent an additional laparotomy, in which macroscopic analysis was performed.

### Surgical procedure

The animals were anaesthetized with an intraperitoneal injection of 50 mg/kg ketamine hydrochloride and 20 mg/kg xylazine hydrochloride.

**Figure 1:**
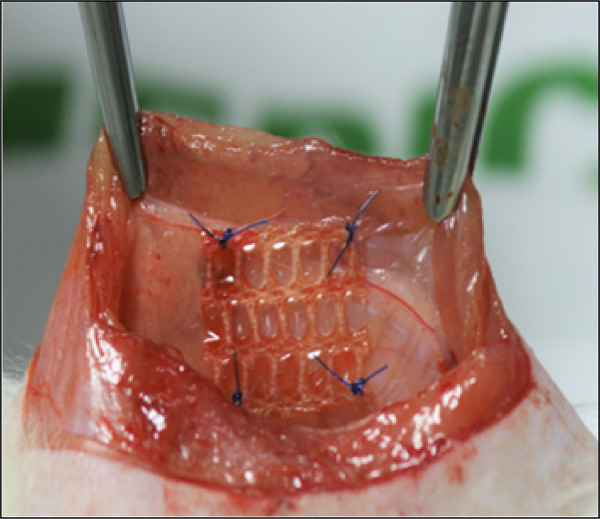
Coated mesh fragment fixed to the abdominal wall.

Source: Elaborated by the authors.

**Figure 2:**
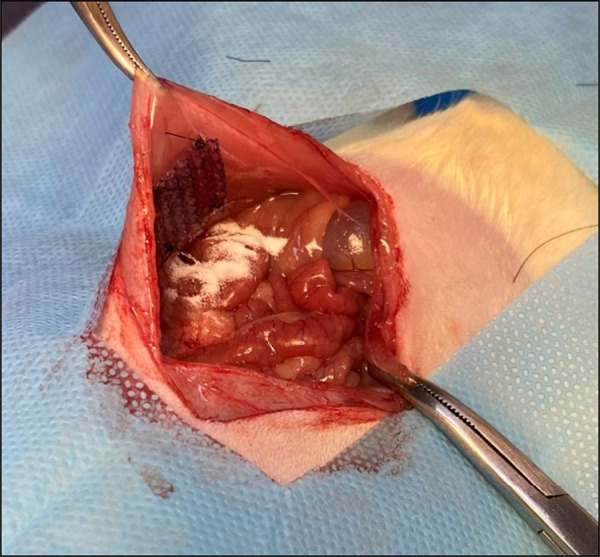
Mesh plus starch and carboxymethylcellulose in the abdominal cavity on the right side.

Source: Elaborated by the authors.

After shaving and antisepsis, an 8-cm long incision was made in the ventral part of the abdomen, involving the skin, aponeurosis, and peritoneum, reaching the abdominal cavity. Thereafter, two fragments of meshes measuring 1.5 × 1,5 cm were interposed and fixed to the abdominal wall 1 cm from the midline, with four simple stiches using 4-0 polypropylene (Fig. 1). Randomly, one side of the abdominal cavity received the topical administration of 0.15 g of SC (Fig. 2). Finally, the abdominal wall was sutured using a 3-0 nylon thread with continuous sutures.

After complete post-anesthesia recovery, the animals were placed in appropriate cages, with a maximum of three animals per cage, being offered water and food ad libitum until the next surgical procedure.

### Euthanasia of the animals and macroscopic post-mortem examination

On the 14th postoperative day (POD), the animals were euthanized using a CO_2_ chamber. Then, necroscopy was performed, and the macroscopic aspects related to the mesh were observed. The degree of adhesions between the organs and meshes (adhesion scoring), area of adhesion between the surface of the mesh and intraperitoneal structures (adhesion coverage on the mesh surface), and integration of the mesh into the abdominal wall (tissue integration score) were evaluated^10^,^29^,^30^ (Tables 1–3).

**Table 1:** Adhesion scoring.

Score	Characteristics
0	Without adhesions
1	Flimsy adhesions: easily removed with blunt dissection and result in limited bleeding.
2	Intermediate adhesions: removed with more aggressive blunt dissection or little sharp dissection, result in moderate bleeding and good plane of dissection present.
3	Firm adhesions: removed only with sharp dissection, bleeds heavily and no plane of dissection present.

Source: Elaborated by the authors.

**Table 2:** Assessment of area of adhesion coverage on the mesh surface.

Adhesion (%)	Grade
0	0
1–25	1
25–50	2
50–75	3
> 75%	4

Source: Elaborated by the authors.

**Table 3:** Tissue integration score.

A	Integration of more than 70 per cent of mesh surface
B	Integration of up to 70 per cent of mesh surface area
C	Moderate integration; no tissue ingrowth through perforation holes and less than 50 per cent of mesh surface integrated

Source: Elaborated by the authors.

### Statistical analysis

The variables were described as absolute and relative percentage frequencies. The hypothesis of equal location measures was tested using the Mann-Whitney’s test. The rank-biserial correlation effect size was estimated to evaluate the size of the observed differences, which evaluates the percentage of evidence favorable to a given hypothesis. The study considered a 5% significance level and used the R Core Team 2022 software (Version 4.2.0).

## Results

One animal died on the nineth POD and another on the 12th POD due to undetermined causes. The mesh used with and without topical SC was evaluated regarding the percentage of surface affected by adhesions, the degree of intensity of the adhesions formed, and the degree of incorporation of the mesh into the abdominal wall on the 14th POD (Table 4).

**Table 4:** Results of adhesions and integration on 14th postoperative day.

Variables evaluated		14 POD		p-value*	R
		Mesh + SCn (%)	Meshn (%)		
Adhesion					
	Yes	38 (100,0)	38 (100,0)	1,000	0,000
	No	0 (0,0)	0 (0,0)		
Area of adhesion (%)					
	0	2 (5,3)	1 (2,6)	0,001	-0,384
	1–25	27 (71,1)	16 (42,1)		
	25–50	4 (10,5)	3 (7,9)		
	50–75	4 (10,5)	7 (18,4)		
	> 75	1 (2,6)	11 (28,9)		
Adhesion scoring					
	Flimsy	29 (76,3)	21 (55,3)	0,039	-0,231
	Intermediate	6 (15,8)	8 (21,1)		
	Firm	3 (7,9)	9 (23,7)		
Tissue integration					
	A	36 (94,7)	37 (97,4)	0,579	0,025
	B	2 (5,3)	0 (0,0)		
	C	0 (0,0)	1 (2,6)		

POD: postoperative day; SC: starch and carboxymethylcellulose; N: absolute frequency; %: relative percentage frequency;

* Mann-Whitney’s test;

R: Rank biserial correlation. Source: Elaborated by the authors.

In all cases, the results showed some degree of adhesion with or without SC, even with coated meshes.

**Figure 3:**
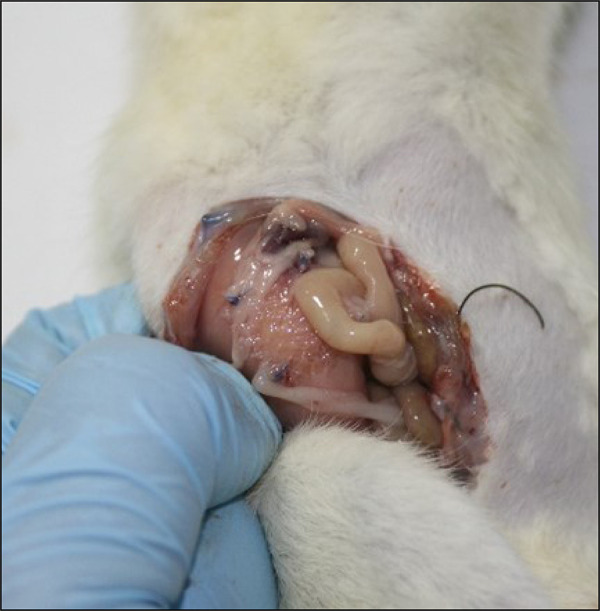
Adhesions between bowel and mesh (25–50% of their surface affected).

Source: Elaborated by the authors.

As for the percentage of the mesh area affected by adhesion, two (5.3%) animals with mesh and SC and one (2.6%) only with mesh had 0% of their surface affected, as they had adhesions only on the mesh edges; 27 (71.1%) animals on the experimental side and 16 (42.1%) on the control side had between 1–25% of their surface affected; four (10.5%) animals on the experimental side and three (7.9%) on the control side had between 25–50% of their surface affected (Fig. 3); four (10.5%) animals on the experimental side and seven (18.4%) on the control side had between 50–75% of their surface affected; and one (2.6%) animal had > 75% adhesion on the experimental side and 11 animals (28.9%) on the control side. The results for the % of mesh surface affected by adhesions (p = 0.001) were significant, with evidence of a higher % adhesion surface on the control side (R = -0.384).

Another variable analyzed was the adhesion intensity score, which showed that 29 (76.3%) animals had flimsy adhesions between the mesh and the intra-abdominal structures on the experimental side, and 21 (55.3%) on the control side. Intermediate adhesions were present in six (15.8%) animals on the experimental side and eight (21.1%) on the control side. Firm adhesions were present in three (7.9%) animals on the experimental side and nine (23.7%) on the control side. The intensity of adhesions formed also showed significant results (p = 0.039), with evidence of firmer adhesions in the control group (R = 0.231).

**Figure 4:**
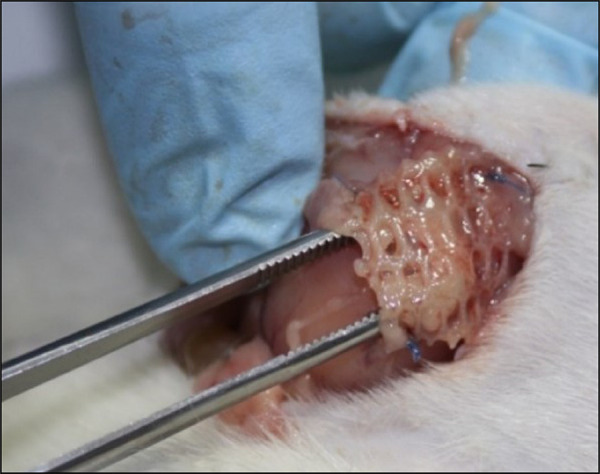
Mesh with less than 50 per cent of mesh surface integrated.

Source: Elaborated by the authors.

SC presented no significant results regarding the integration between the mesh and the abdominal wall (p = 0.579), with 36 (94.7%) animals on the experimental side and 37 (97.4%) on the control side being classified as A; two experimental animals (5.3%) and no control classified as B; and only one (2.6%) control animal classified as C (Fig. 4).

## Discussion

As mesh adhesions are one of the main complications and cause of concern when using the IPOM technique, surgeons seek ways to reduce or stop these adhesions. Some surgical techniques avoid the use of intraperitoneal meshes and access retromuscular or preperitoneal spaces^31^-^34^. However, these options are not always possible, and IPOM is necessary, especially in urgent surgeries with older and/or obese patients. In addition, these new techniques are more complex, requiring specific training and sometimes the use of robotic platforms^35^. Another way to prevent these adhesions is to use topical agents that act as an additional protective barrier that separates the mesh from adjacent intra-abdominal structures during the healing process in the critical period of adhesion formation^28^.

In the present study, despite using a coated mesh, all animals showed some degree of adhesion. As for the percentage of the mesh area affected by adhesion, two animals (5.2%) on the experimental side and one (2.6%) on the control had 0% of the surface affected, as they had adhesions only on the mesh edges. The other animals showed adhesions on the mesh surface with or without SC. Some experimental and even clinical studies demonstrated that, even with the protective barrier, these intraperitoneal meshes present different degrees of adhesions with intra-abdominal structures^9^-^13^,^36^. For this reason, ways to mitigate this complication and its clinical repercussions are being studied. In the present study, the SC protective barrier did not reduce the incidence of adhesions, but other aspects evaluated, such as adhesion intensity and surface area, were decreased after its use.

The meshes coated with a protective barrier have different materials on their visceral surface that allow contact between the mesh and intra-abdominal organs. These materials include polyvinylidene fluoride, polylactic acid, oxygenated regenerated cellulose, polyglycolic acid, collagen, and chitosan^37^,^38^. The Symbotex mesh used in the experiment has collagen as a protective material. The use of a second protection mechanism, as in the experiment, added to the one already present on the mesh, may have synergistically acted and offered extra protection that resulted in lower intensity and smaller area of adhesion. The SC barrier protects the intra-abdominal organs from mechanical injuries caused by contact with the mesh, thus decreasing adhesion formation, since less contact and trauma decrease tissue damage and increase vascular permeability with fibrinogen release and fibrin formation^39^. Other meshes with different protective materials associated with the topical use of SC can be tested in future studies to clarify whether this probable added protection effect also occurs with other materials.

The product used in the experiment is available in the Brazilian market under the commercial name Adhesion STP. It is marketed in the form of powder or gel, with packages of 1, 3, or 5 g and with the possibility of laparoscopic application. This study used the powder presentation as it can be applied to a larger area and it is easier to use. Another brand like the one used in the study is the 4DryField (4DF), which contains only starch and no carboxymethylcellulose and it is not available in Brazil, but it is used in Europe. This product is also available as powder and in 1-, 3-, 5-, and 9-g packages. Arista is another product containing the plant polysaccharide starch and it is also available as powder and in presentations of 1, 3, and 5 g.

This experiment used a total of 6 g of SC (two 3-g bottles), divided equally among 40 rats, totaling 0.15 g of the product in each animal, with the contralateral side of the studied abdomen being the control. Such volume was determined based on the total coverage of half of the abdominal cavity of each animal, with SC being sufficient to cover the contact area between the mesh and the intracavitary organs, respecting the proposed dose of up to 1 g/kg^28^. It is not clear whether the use of larger volumes can further reduce the adhesion intensity and area. Therefore, further studies evaluating this aspect are necessary. Larger volumes than those available in current presentations may be necessary for the protective effect to occur in clinical practice, especially when larger meshes are used, respecting its tolerability.

There are no studies in the literature evaluating the systemic effects of SC in the form of ASTP. There is also no mention of side effects in its instructions for use. 4DF, in turn, brings a package insert warning regarding the possibility of an inflammatory reaction with increased C-reactive protein after its use. However, a retrospective study evaluated systemic inflammatory effects, and this product was considered safe as it did not induce a significant systemic inflammatory response in patients using between 3–5 g of 4DF^40^. Another experimental study also evaluated the tolerability of this same presentation and concluded that the product had excellent biocompatibility with in-vitro analysis, showing no cytotoxicity or tumor cell growth. This analysis demonstrated that a dose of up to 1.09 g/kg is well tolerated and degraded after a few days^28^. Experimental foreign body reaction was also considered minimal^41^. Although these are plant-derived products with good biocompatibility and low risk of triggering allergic reactions, future experimental studies are also needed to assess the systemic effects and tolerability of SC in the form of ASTP.

Plant polysaccharides have been used to avoid intra-abdominal adhesions after conventional surgery. On an experimental basis, compared to other non-adherent agents, it significantly reduced the incidence and severity of adhesions after laparotomies^42^. It has also been studied in case series, with evidence of decreased new adhesion formation with topical use in a two-year follow-up^20^.

Another clinical study evaluated 20 women undergoing re-laparotomies after using 4DF, showing that 18 of them had effective adhesion prevention with topical use of 3–15 g of the product^19^. As for its specific ability to prevent or reduce adhesions between intraperitoneal meshes and intra-abdominal structures, only one experimental study using 4DF starch is available in the literature^23^. That study evaluated 20 rats using the gel and powder presentations, having as a control group study previously published in the literature. Unlike the present study, that study used conventional meshes without a protective barrier. Even so, the product significantly reduced adhesions, especially in powder form. In the present study, we chose to use coated meshes, since they are the meshes used in clinical practice, which have also resulted in adhesion formation. The Arista plant polysaccharide also demonstrated decreased adhesion formation in an experiment with its use as a hemostatic agent^43^. Starch has a synergistic effect when associated with carboxymethylcellulose, providing additional capacity to reduce intra-abdominal adhesions and abscess formation, as demonstrated in an experimental study^25^. No other studies prior to this one used starch associated with carboxymethylcellulose (Adhesion STP) to avoid adherence to coated meshes.

As for the intensity of the adhesions evaluated in the study, there were mesh adhesions with the intestine and liver on the side of the mesh without SC (Fig. 3). A higher percentage of firm adhesions was also observed on the control side compared to the experimental group. In this group, among the nine firm adhesions, three involved the intestine and liver. This finding demonstrates that complications could be significant if this concept is extrapolated to humans.

As for the percentage of the surface area of the mesh affected by adhesions, most of the adhesions on the SC side scored 1–25%, and only one animal had adhesions in > 75% of the mesh. In the sided without SC, the number of animals with adhesions decreased in the score between 1–25% and increased in the score between 5–75% and > 75%, demonstrating protective effect of SC in the area affected by adhesions.

As for incorporation, the purpose of this study was to assess whether the topical use of SC could interfere in this process, but there was no statistically significant difference, i.e., its use did not interfere with the mesh incorporation into the abdominal wall. Only one animal on the side without SC had < 50% incorporation. This is important because the proper incorporation of the mesh into the abdominal wall is crucial for the outcome of hernia surgeries. Thus, the product could not interfere with the biological process of fibrous tissue formation.

Since this is the only study evaluating the effect of ASTP, with another experimental study evaluating the effect of 4DF in preventing intraperitoneal mesh adhesions, different experimental studies with larger samples, different dosages, other meshes, and even comparing the available brands are needed before we can propose the clinical use of this product.

## Conclusion

The topical use of the product composed of SC experimentally decreased the intensity of adhesions and the surface area of the mesh affected by adhesions with intra-abdominal structures. There was no negative interference of the product in the incorporation of the mesh into the abdominal wall.

## Data Availability

The data will be available upon request
